# Hybrid telerehabilitation approach for patellofemoral pain management in South African runners: a feasibility case series

**DOI:** 10.3389/fdgth.2025.1678382

**Published:** 2025-10-07

**Authors:** Eugene Nizeyimana, Onele Malunga, Dawn Ernstzen, Quinette Louw

**Affiliations:** Department of Health and Rehabiliation Sciences, Stellenbosch University, Stellenbosch, South Africa

**Keywords:** feasibility, hybrid rehabilitation, in-person rehabilitation, patellofemoral pain, runners, South Africa, telerehabilitation, WhatsApp video consultations

## Abstract

**Background:**

Patellofemoral pain (PFP) is a prevalent condition in sports medicine, with rising incidence as sports competitions become increasingly popular. South Africa's healthcare system faces substantial challenges in delivering rehabilitation services due to geographical constraints, limited resources including professional shortages, and inadequate access to specialised musculoskeletal care. This study evaluated the feasibility of implementing a hybrid telerehabilitation program combining face-to-face sessions with WhatsApp video consultations for managing PFP in South African runners.

**Methods:**

A feasibility case series was conducted with five runners aged 25–39 years with PFP duration ≥6 weeks, recruited from Johannesburg. The 6-week intervention comprised an initial in-person assessment, weekly WhatsApp video consultations, and bi-weekly face-to-face sessions. Primary feasibility outcomes included recruitment success, session adherence, and acceptability measured using the Telehealth Usability Questionnaire (TUQ). Secondary clinical outcomes assessed pain intensity (Numerical Pain Rating Scale) and functional status (Anterior Knee Pain Scale).

**Results:**

Recruitment targets were fully achieved with 100% adherence to all scheduled sessions. Participants demonstrated high exercise compliance and good acceptability scores (mean TUQ 5.9/7), though participants expressed a preference for in-person consultations. Significant clinical improvements were observed, with pain scores decreasing from 3.8 to 0.6 and functional scores improving from 79.6 to 94.0 over six weeks.

**Conclusion:**

Hybrid telerehabilitation demonstrated feasibility and preliminary effectiveness for PFP management in South African runners, achieving excellent adherence rates and clinically meaningful improvements in pain and function. This approach shows promise for addressing healthcare delivery challenges in resource-constrained settings.

## Introduction

Patellofemoral pain (PFP) is a prevalent condition in sports medicine, with rising incidence as sports competitions become increasingly popular (1). Characterised by anterior knee pain, PFP presents notable clinical challenges due to its chronic nature and the potential for long-term musculoskeletal complications. If not adequately managed, PFP may predispose individuals to patellofemoral joint osteoarthritis, thereby exacerbating disability and reducing quality of life (QoL) (2). The chronic nature of PFP presents a particular challenge, with studies indicating that over 90% of affected individuals continue experiencing symptoms for years following initial diagnosis (3).

South Africa's healthcare system struggles with chronic musculoskeletal conditions like PFP due to professional shortages, limited rehabilitation services, and extended waiting lists, particularly in rural and underserved areas. While Johannesburg has better infrastructure, professional shortages persist. In addition, Johannesburg is very big with few healthcare centres, making TR valuable for reducing travel time.

These barriers are further exacerbated by the nation's quadruple burden of disease, characterised by a convergence of communicable and non-communicable diseases, high maternal and child morbidity, and trauma-related conditions (4). The increasing prevalence of chronic illnesses, coupled with socioeconomic constraints, places additional strain on existing healthcare resources, necessitating innovative and cost-effective solutions to improve rehabilitation access and patient outcomes.

Telerehabilitation (TR) has emerged as a viable alternative for addressing healthcare delivery challenges by leveraging digital communication technologies to provide remote rehabilitation services. TR encompasses virtual assessments, treatment sessions, preventive care, patient education, and therapeutic guidance, delivered through synchronous modes (real-time interactive communication between patient and provider), asynchronous modes (store-and-forward transmission where patient information is reviewed and responded to at different times), or blended/hybrid modes (integrated combination of virtual and in-person care delivery), ensuring continuity of care beyond traditional face-to-face settings (5–7). This approach mitigates logistical barriers, reduces travel distances, appointment waiting times, and associated costs, including income loss due to missed work (8–10).

Previous studies have indicated that TR yields significant improvements in pain reduction, range of motion, and overall patient satisfaction in patients with musculoskeletal disorders (10, 11). Wang et al. (8) reported superior long-term improvements in pain and functionality among individuals receiving TR interventions compared to those receiving no rehabilitation. Specifically for patellofemoral pain (PFP), TR interventions demonstrate effectiveness comparable to traditional in-person rehabilitation, with similar outcomes in pain reduction, functional improvement, and muscle strength enhancement (12–15). Most TR programs adopt hybrid models incorporating initial in-person sessions followed by structured online exercise programs and remote follow-ups, with early intervention being particularly crucial in preventing progression to patellofemoral osteoarthritis and mitigating long-term disability risks (16).

The growing evidence supporting TR underscores its potential as an equitable and accessible rehabilitation modality, particularly in resource-limited settings such as South Africa (17). However, successful implementation requires careful consideration of contextual factors, including digital infrastructure, device accessibility, digital literacy levels, cultural acceptability, and regulatory frameworks governing telehealth services (9, 18). Additionally, healthcare professionals must acquire the necessary skills for effective remote service delivery, including proficiency in virtual assessment techniques and adherence to medical and legal telehealth standards (9, 19). Despite growing global evidence supporting TR, South Africa lacks locally adapted TR programs for PFP management (15).

This study aimed to assess the feasibility of implementing a hybrid TR program for physiotherapeutic management of runners with PFP in South Africa, evaluating its practicality, acceptability, and potential effectiveness within the country's unique healthcare context. The findings will provide critical insights for healthcare practitioners delivering TR services while informing implementation strategies and policy development for resource-constrained settings. As a feasibility study examining hybrid TR for PFP patients in South Africa, the results will establish foundational evidence regarding feasibility, acceptability, and preliminary effectiveness that will directly inform the design, methodology, and implementation protocols for larger-scale randomised controlled trials and multi-site studies in similar low- and middle-income country contexts.

## Materials and methods

### Ethics

Ethics approval for this study was granted by the Health Research Ethics Committee of Stellenbosch University (S22/11/228_Sub Study_N19/05/063).

### Study design

This feasibility study employed a descriptive case series design with five South African runners to examine the implementation and preliminary outcomes of a hybrid TR approach for managing patellofemoral pain. Case series designs are particularly valuable for investigating novel interventions and exploring clinical phenomena in specific populations, providing detailed descriptions of individual patient responses and identifying patterns across cases (20). This design enables documentation of intervention characteristics, patient responses, and implementation considerations without the requirement for control groups, making it ideal for preliminary evaluation of innovative treatment approaches (21). The case series methodology facilitates in-depth exploration of treatment feasibility, patient adherence, and contextual factors that influence intervention delivery in real-world clinical settings.

Given that hybrid TR represents a novel therapeutic approach in the South African healthcare context, particularly for managing musculoskeletal conditions in athletic populations, a feasibility study using a case series design with five participants was deemed appropriate for this preliminary investigation. The small sample size typical of feasibility studies enables detailed case-by-case analysis while providing essential insights into the applicability of hybrid TR within South African healthcare infrastructure, contributing valuable preliminary data to inform the design of larger-scale research initiatives and evidence-based practice development in resource-constrained settings (20, 21).

### Study setting

The initial patient evaluations were conducted at a private outpatient physiotherapy practice in Alberton, Johannesburg, Gauteng Province, South Africa. While the primary researcher performed assessments and delivered TR services from the practice's facilities, participants engaged in remote sessions from self-selected locations meeting two criteria: sufficient space for exercise execution, smart devices and stable internet connectivity. All assessment data were collected at the physiotherapy outpatient department in Alberton.

### Population and sampling

The study population consisted of recreational and competitive runners in Johannesburg, Gauteng, presenting with PFP. Using non-probability consecutive sampling, five participants aged 25–39 years were recruited. Inclusion criteria encompassed both unilateral and bilateral PFP persisting for ≥6 weeks, diagnosed using Leibbrandt and Louw's screening tool (2). The Leibbrandt and Louw screening tool is an evidence-based diagnostic checklist for anterior knee pain (AKP) developed to address the lack of standardized diagnostic criteria for this condition that commonly limits daily activities. The tool includes criteria for pain location, age, symptom duration, aggravating factors, and functional tests, with squatting showing the highest sensitivity and a recommended cluster approach using two of three positive findings from squatting, isometric quadriceps contraction, and patella border palpation. The tool's validity is based on two high-quality systematic reviews (scoring 8/10 methodologically) encompassing nine diagnostic studies (2).

Exclusion criteria comprised conditions such as osteoarthritis, rheumatoid arthritis, patellar fractures/subluxation/dislocation, fat pad impingement, bursitis, Osgood-Schlatter disease, intra-articular pathology, patellar tendinitis, and referred pain from the lumbar spine or hip.

## Outcome measures

### Feasibility assessment methods

The feasibility assessment examined recruitment success, intervention adherence, and program acceptability. Table 1 highlights feasibility assessment framework.

**Table 1:** Feasibility outcome measurement framework.

Outcomes	Operational definition	Target threshold	Measurement methods
Recruitment	Number of eligible participants enrolled per month	Minimum of 2 participants per month	Documented in standardised study records by the research coordinator
Attendance	Completion rates for both modalities: (1) WhatsApp virtual sessions and (2) face-to-face clinical appointments	Full participation in scheduled protocol: 4 online sessions and 3 face-to-face sessions	Systematically recorded in the study database
Adherence	Adherence to the prescribed home exercise regimen	Minimum exercise frequency of three sessions weekly per participant	Self-reported through structured weekly exercise diaries with verification during clinical contacts
Acceptability	Participant receptiveness to the hybrid intervention delivery model	Complete TUQ data from all study participants	Analysis of TUQ responses with additional qualitative feedback collection

### Recruitment metrics

The recruitment process for this feasibility study commenced with the dissemination of the study advertisement via email to four running clubs located in the South and East regions of Johannesburg, Gauteng, on February 21, 2023. Simultaneously, physical posters for advertising the study were placed in the physiotherapy department. The recruitment rate was systematically monitored by documenting the number of eligible participants enrolled per specified period. All recruitment data were recorded in a centralised study database to facilitate ongoing assessment of recruitment patterns.

### Intervention adherence

Intervention adherence was systematically evaluated across multiple domains. In-person protocol compliance was documented through participant attendance at scheduled face-to-face sessions. Remote intervention adherence encompassed: (1) participation in weekly physiotherapist-supervised WhatsApp video consultations, and (2) completion of prescribed therapeutic exercises.

Exercises were individualized based on participants’ functional capacity and pain responses during standardized movement assessments. Exercise prescription integrated findings from functional assessments evaluating both pain provocation and movement quality during squats, kneeling, stair descent, isometric quadriceps contractions, and patellofemoral compression. Participants rated pain intensity using NPRS (0–10) during these activities to establish baseline functional capacity, identify movement-specific limitations, and create individualized progressions balancing therapeutic load with tolerability. Pain intensity was assessed during five standardized provocation tests: squats, kneeling, stair descent, isometric quadriceps contractions, and patellofemoral compression. At each assessment timepoint (baseline, 3 weeks, 6 weeks), individual participant mean scores were calculated by averaging their NPRS ratings across all five provocation tests. The overall group mean score was then derived by averaging these individual participant means. Group mean pain scores demonstrated progressive improvement, decreasing significantly from 3.8 at baseline to 3.0 at three weeks and 0.6 at six weeks, indicating clinically meaningful pain reduction throughout the intervention period.

Recognizing that chronic patellofemoral pain often precludes completely pain-free exercise, the intervention adopted a capacity-based approach rather than strict pain avoidance. Exercise prescription prioritized functional movement quality and ability to perform exercises with acceptable pain levels (defined as not exceeding baseline provocation pain by >2 points), acknowledging the biopsychosocial nature of chronic pain where complete pain elimination is often unrealistic.

Participants with higher pain intensity and movement limitations (AB1: 6/10, GH4: 5/10) received foundational exercises targeting movement re-education and load tolerance. Those demonstrating moderate pain with better movement quality (EF3, IJ5: 3/10) progressed to dynamic strengthening exercises. The participant showing minimal pain with good movement competency (KL6: 2/10) received advanced functional exercises.

To address confounding factors like regression to the mean and day-to-day pain variability, exercise progression was continuously monitored through weekly WhatsApp consultations with real-time NPRS assessment and functional capacity evaluation, allowing dynamic modification based on current functional status rather than rigid adherence to initial baseline scores.

### Acceptability

The Telehealth Usability Questionnaire (TUQ) was administered at program completion to evaluate acceptability of the hybrid intervention approach, providing standardized measurement of user experience with telehealth components.

### Secondary outcome measures

Three validated instruments were utilized: the Numerical Pain Rating Scale (NPRS), Anterior Knee Pain Scale (AKPS), and Telehealth Usability Questionnaire (TUQ).

#### Numerical pain rating scale (NPRS)

The NPRS, an 11-point scale (0 = “no pain” to 10 = “worst pain”) with excellent reliability and validity (22), assessed pain during functional movements, established baseline capacity, identified movement-specific limitations, and guide individualized exercise progressions. Acceptable pain levels were defined as not exceeding baseline provocation pain by >2 points. Weekly real-time NPRS monitoring enabled dynamic exercise modifications based on current functional status.

The Anterior Knee Pain Scale (AKPS) comprises 13 self-report categories evaluating the functional impact of PFP on activities of daily living. Scores range from 0 to 100, with lower scores indicating greater pain and disability. Ittenbach et al. (23) reported high reliability and validity for the AKPS, while Watson et al. (24) documented strong test-retest reliability and moderate responsiveness to clinical change in PFP patients.

The Telehealth Usability Questionnaire (TUQ) assesses telehealth system usability across multiple domains: usefulness, ease of use, effectiveness, reliability, and user satisfaction. Developed for various telehealth modalities including videoconferencing, the TUQ employs a 7-point Likert scale (1 = “strongly disagree” to 7 = “strongly agree”), with higher scores indicating more positive responses. Parmanto et al. (25) reported good to excellent reliability across user factors and strong content validity, making it suitable for evaluating hybrid healthcare delivery approaches.

### Procedure

Participant recruitment followed dual pathways: direct physiotherapist referrals and self-referrals responding to advertisements. The strategy targeted socioeconomically diverse runners in the Johannesburg metropolitan area, Gauteng Province, employing multiple recruitment channels to maximise participant reach, consistent with established clinical research practices (26). Strategic advertisement placement occurred in hospitals and physiotherapy practices in South and East Johannesburg, particularly in outpatient departments.

Electronic recruitment utilised targeted email campaigns to running clubs and Parkrun organisations in Johannesburg, identified through systematic online searches. Recruitment emails contained comprehensive study information and consent forms. Club administrators received detailed materials, including advertisement posters for member dissemination. After obtaining consent, all candidates underwent preliminary eligibility screening using a modified evidence-based diagnostic tool (2). Recruitment through advertisements in the hospital and physiotherapy department generated interest from 13 potential participants (8 from the hospital and 5 from the physiotherapy department). Following eligibility screening, individuals were excluded based on predetermined criteria: 4 presented with osteoarthritis, 2 with rheumatoid arthritis, and 2 with patellar tendinitis. Consequently, 5 participants satisfied the inclusion criteria and proceeded to baseline assessment.

### Intervention schedule

The hybrid intervention alternated session types by week. WhatsApp video sessions were scheduled in weeks 1, 2, 4, and 5, while face-to-face sessions occurred in weeks 1, 3, and 6. Week 1 included both session types for baseline assessment. Sessions were mutually exclusive in subsequent weeks.”

All outcome measurements (NPRS, AKPS at baseline, 3-week, and 6-week timepoints, and TUQ at program completion) were conducted face-to-face during scheduled in-person sessions at the physiotherapy practice to ensure measurement consistency and reliability.

### Data analysis

Data were analysed using IBM SPSS Statistics Version 29. Given the small feasibility sample (*n* = 5), descriptive statistics were the primary analytical approach, with no inferential testing performed due to insufficient statistical power.

Recruitment metrics were comprehensively evaluated to assess the study's feasibility in enrolling participants from the target population. Total enrolment was documented alongside demographic distributions presented as frequencies and percentages, with detailed tracking of referral sources to identify the most effective recruitment channels. Recruitment efficiency was quantified using the standardized formula proposed by Jaques et al. (27), calculated as the number of enrolled participants divided by the product of recruitment sites and recruitment duration in months, yielding a recruitment rate of 1.176 participants per site per month.

Following successful enrolment, intervention adherence was systematically monitored through multiple complementary approaches. Exercise program compliance was quantified as the percentage of prescribed sessions completed, derived from participant self-reported weekly logs maintained throughout the study duration. The analysis employed two primary adherence metrics to capture the hybrid nature of the intervention: attendance rates at mandatory in-person sessions and participation frequency in WhatsApp-facilitated virtual sessions. Mean values, standard deviations, and ranges were computed for both delivery modalities to provide comprehensive adherence profiles across different intervention components.

Participant acceptability of the hybrid TR approach was assessed using the Telehealth Usability Questionnaire (TUQ), which provided quantitative measures of user experience and satisfaction with the intervention delivery methods. Mean scores and standard deviations were calculated across all questionnaire domains and individual statements to identify specific aspects of the intervention that were most and least acceptable to participants, thereby informing future implementation strategies and intervention refinements.

### Pre-Post intervention analysis

The preliminary efficacy was evaluated using validated outcome measures at baseline (T0), mid-intervention (T1; week 3), and post-intervention (T2; week 6). For both NPRS (0–10) and AKPS (0–100), individual participant change scores were calculated for each interval (T1-T0, T2-T0, T2-T1). Mean change scores with standard deviations were computed to quantify intervention effects. Individual response patterns were examined through case-by-case trajectory analysis across time points. Clinical significance was assessed using established minimal clinically important difference thresholds: ≥2-point reduction for NPRS (28) and ≥10-point improvement for AKPS (29). TUQ scores were interpreted using descriptive analysis, as no established cut-off points exist for this instrument (25). Following the approach used in previous studies, scores above 5.0 on the 7-point Likert scale were considered indicative of positive usability, consistent with mean scores reported in similar telehealth feasibility studies (30). No statistical significance testing was conducted given the descriptive feasibility design and sample size limitations.

## Results

### Primary outcomes (feasibility)

Participant Demographics: The study comprised five participants, with an age range of 25–39 years. The sample included three female and two male participants, all of whom were active runners based in Johannesburg, Gauteng. Of the participants, two engaged in weekly 5 km runs, while the remaining three ran distances of more than 10 kms per week. Table 2 provides a detailed summary of participant demographics, including gender, age, running distance, affected knee side, and recruitment channel.

**Table 2:** Participant demographics.

Participants	Gender	Age	Average running distance per week	Affected knee side	Recruitment channel
GH4	Male	31	5 km	R	Physio OPD
EF3	Male	25	>10 km	R	Email
IJ5	Female	35	>10 km	R	Email
AB1	Female	39	5 km	R	Physio OPD
KL6	Female	31	>10 km	L	Email

GH4 = participant one, EF3 = participant two, IJ5 = participant three, AB1 = participant four, KL6 = participant 5.

### Study participant recruitment rate

The recruitment process for this feasibility study commenced with the dissemination of the study advertisement via email to four running clubs located in the South and East regions of Johannesburg, Gauteng, on February 21, 2023. Simultaneously, physical posters were placed in the physiotherapy department. The first response from a potential participant was received via email on February 25, 2023, and the final response was recorded on May 26, 2023. Of the five eligible participants recruited, two were identified through responses to the advertisement in the physiotherapy department, while the remaining three were recruited through email outreach to running clubs. The recruitment period lasted 13 weeks and three days, successfully achieving the study's target of recruiting at least one participant per month, culminating in a total of five participants by the end of the four-month recruitment window.

### Adherence to online WhatsApp TR sessions

**Figure 1:**
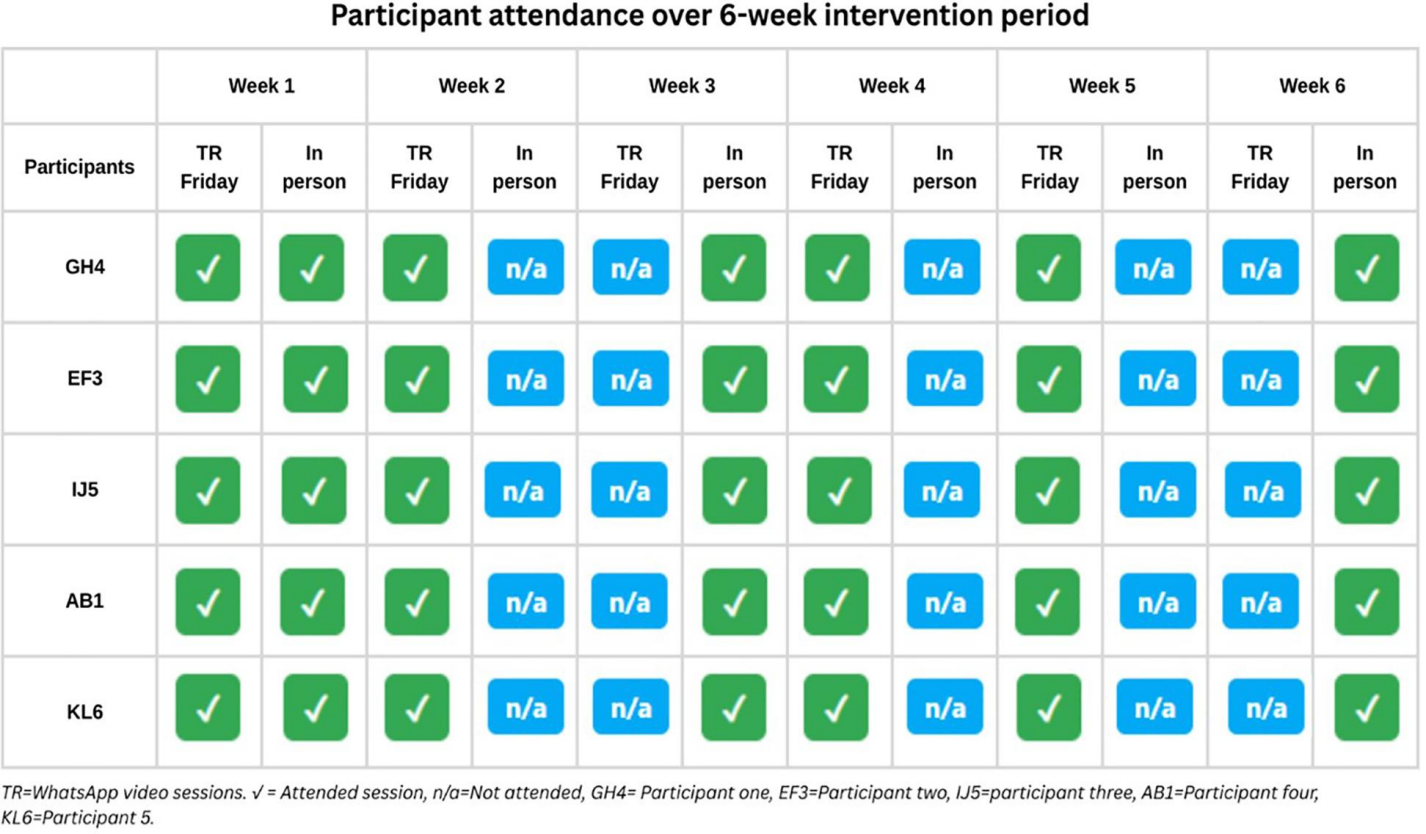
The attendance of each participant over the six-week intervention period.

Session attendance was excellent across all participants throughout the 6-week intervention period. All participants achieved 100% attendance for both scheduled WhatsApp video sessions (weekly) and face-to-face sessions (weeks 1, 3, and 6). Home exercise adherence, defined as completing prescribed exercises at least three times per week, was also 100% across all participants. Both session attendance and home exercise adherence demonstrated high compliance rates, supporting the feasibility of the hybrid telerehabilitation approach. Figure 1 presents the detailed session attendance of each participant over the six-week intervention period.

**Figure 2:**
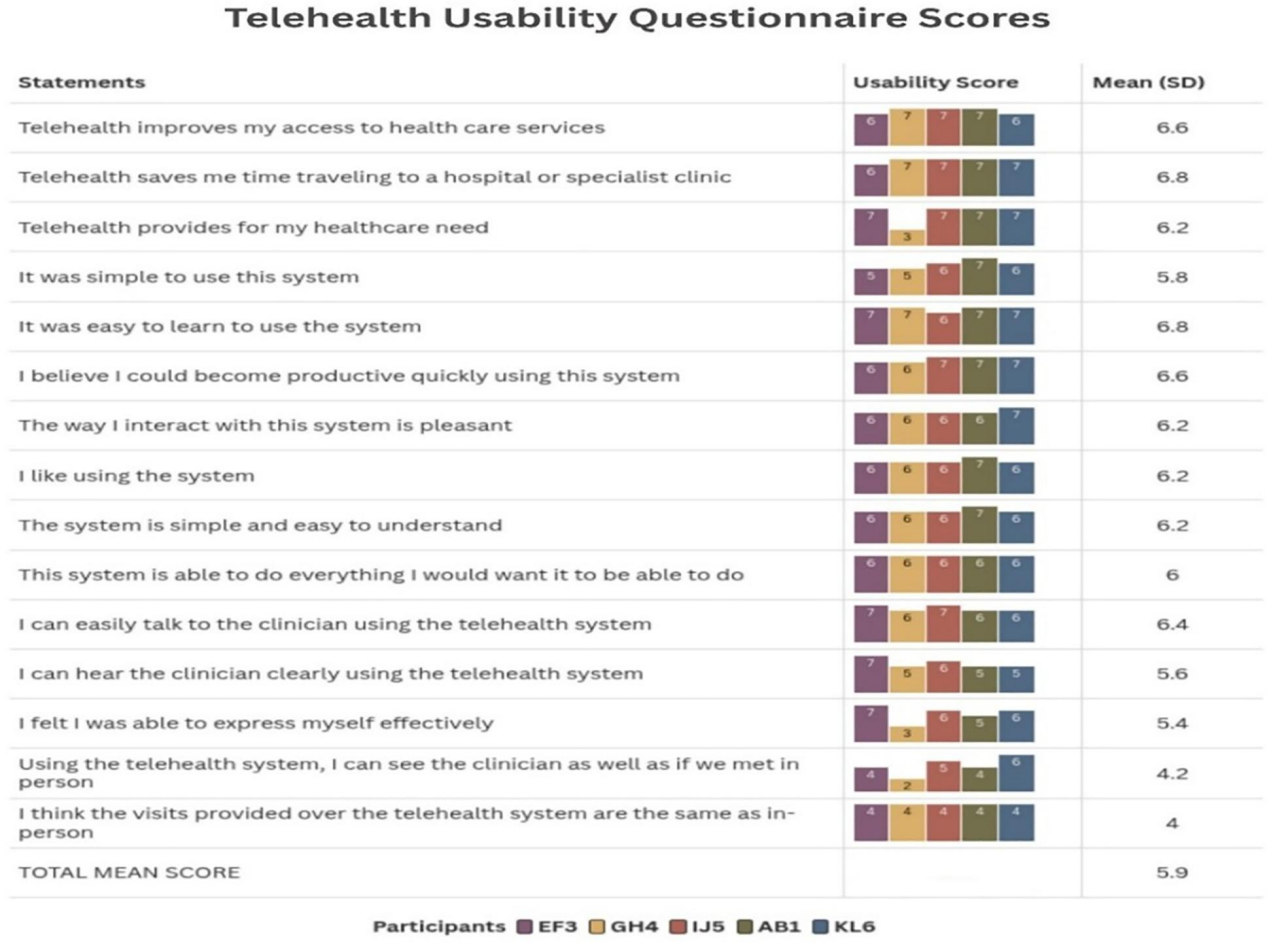
Individual and mean telehealth usability questionnaire scores.

The Telehealth Usability Questionnaire demonstrated high overall satisfaction with a total mean score of 5.9. Participants rated time-saving benefits and ease of learning highest (6.8 each), while visual presence compared to in-person visits scored lowest (4.0–4.2). All participants showed consistently positive responses across usability domains. Figure 2 provides a detailed breakdown of individual and mean TUQ scores.

### Secondary outcomes (preliminary effectiveness

This section presents the results from two key assessments used to evaluate preliminary intervention outcomes: the Numerical Pain Rating Scale (NPRS) and Anterior Knee Pain Scale (AKPS). These measurements, collected at baseline, three weeks, and six weeks, provide insights into pain intensity and functional disability improvements following the intervention. Significant improvements were observed in both pain and functional outcomes over the six-week intervention period. Mean NPRS pain scores decreased substantially from 3.8 at baseline to 0.6 at 6 weeks follow-up, while AKPS functional scores improved from 79.6 to 94.0, indicating clinically meaningful improvements in both primary outcome measures.

### Numerical pain rating scale (NPRS)

Participants rated pain intensity using the NPRS (0–10) during standardized provocation tests including squats, kneeling, stair descent, isometric quadriceps contractions, and patellofemoral compression. All participants experienced pain during squats, kneeling, descending stairs, isometric quadriceps, and compression tests. Stair ascending and patellar palpation provoked pain in 60% of participants; prolonged sitting and patella tilt testing in 40%.

**Figure 3:**
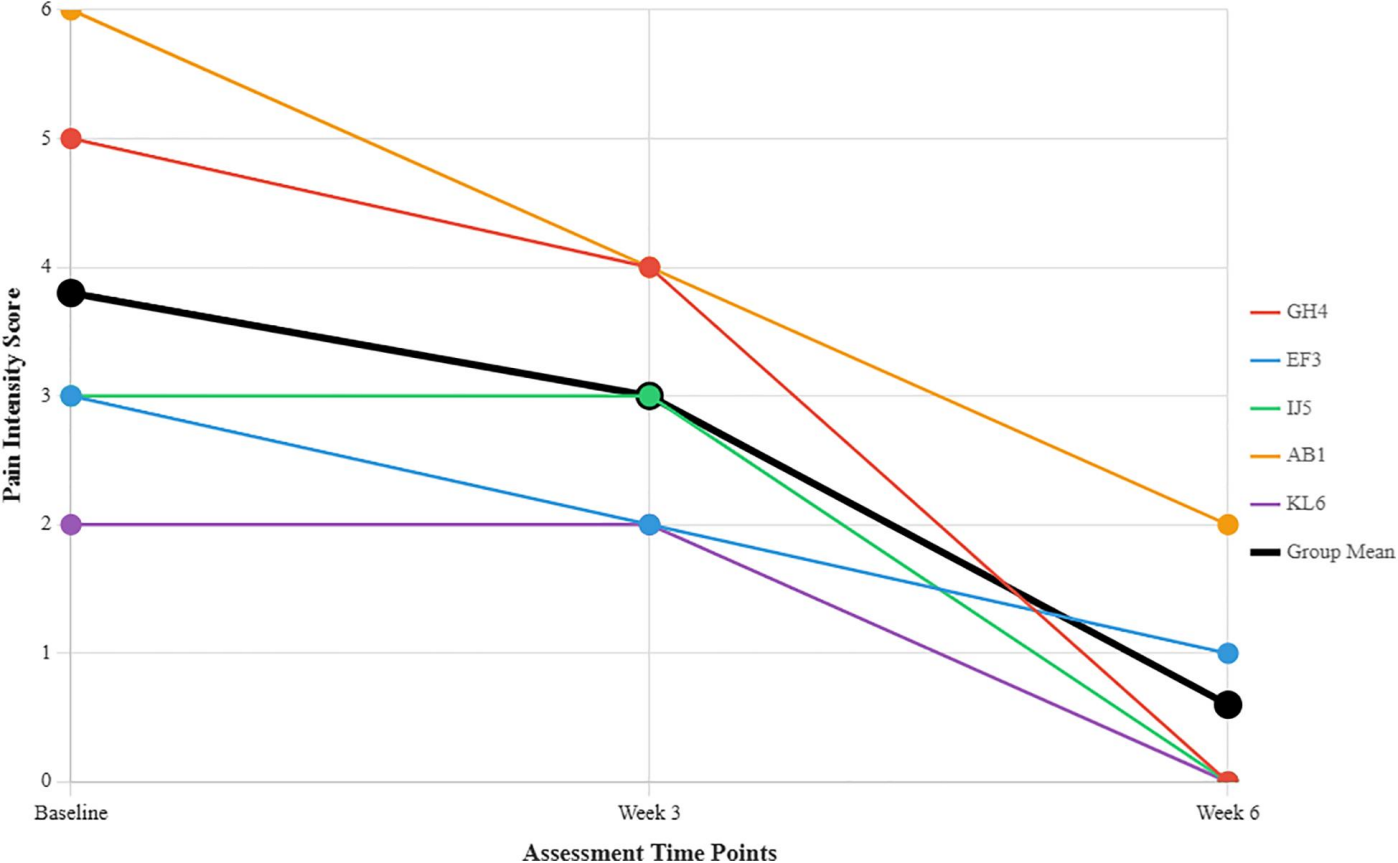
Individual participant pain intensity scores and group mean across three assessment time points. Each coloured line represents an individual participant (GH4 = Participant 1, EF3 = Participant 2, IJ5 = Participant 3, AB1 = Participant 4, KL6 = Participant 5). The thick black line shows the group mean.

At the three-week follow-up, pain reduction was observed in three participants (60%), while two participants (40%) reported no change in pain intensity. By the end of the six-week intervention, all five participants (100%) reported a reduction in pain compared to baseline, with three participants (60%) achieving complete pain resolution. The mean pain score decreased from 3.8 at baseline to 3.0 at three weeks and further to 0.6 at six weeks, demonstrating a clinically significant reduction in pain intensity. Effectiveness outcomes were secondary exploratory measures in this feasibility study. Findings represent preliminary indicators of intervention promise, not definitive efficacy demonstration, requiring larger controlled trials for definitive effectiveness evaluation. Figure 3 shows individual and group mean pain intensity scores across all five provocation tests at three assessment timepoints, demonstrating progressive improvement from baseline (3.8) through three weeks (3.0) to six weeks (0.6).

Key Findings in Figure 3:

• Mean pain intensity decreased significantly from 3.8 at baseline to 0.6 at six weeks, representing a 3.2-point reduction
• All participants showed improvement in pain scores over the 6-week period
• Greatest improvement observed in participant AB1 (6→2) and GH4 (5→0)

### Anterior knee pain scale (AKPS) scores

**Figure 4:**
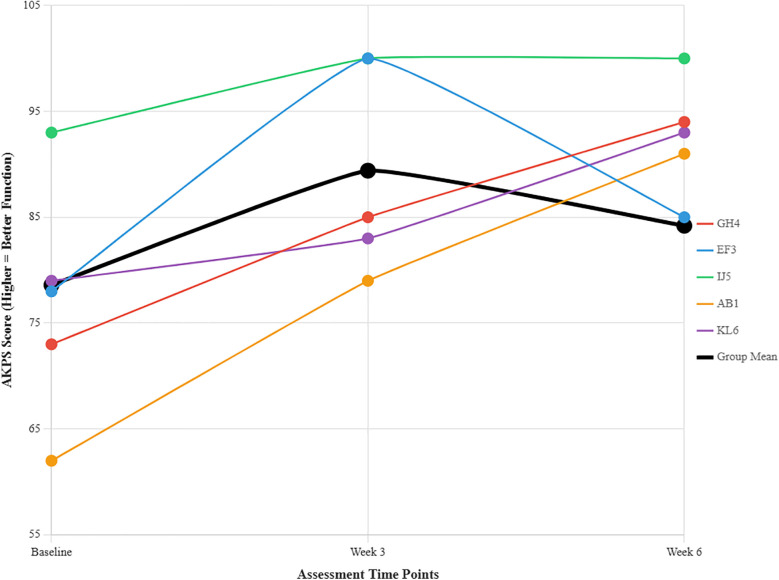
Individual participant anterior knee pain scale (AKPS) scores across three assessment time points. Each coloured line represents an individual participant (GH4 = Participant 1, EF3 = Participant 2, IJ5 = Participant 3, AB1 = Participant 4, KL6 = Participant 5). The thick black line shows the group mean. Higher scores indicate better knee function. Most participants showed peak functional improvement at week 3, with some decline by week 6 while still maintaining gains above baseline.

Functional disability associated with PFP was assessed using AKPS. Baseline AKPS scores ranged from 62 to 93, with a mean of 79.6. A score of 70 indicates moderate disability, with lower scores reflecting greater functional impairment. At the three-week assessment, the mean AKPS score improved to 84.2, and by the six-week follow-up, the mean score further increased to 94. The mean AKPS score improved significantly from baseline (79.6) to six-week follow-up (94), representing a 14.4-point increase indicating substantial functional improvement in patellofemoral pain symptoms. This trend suggests substantial functional improvements following the intervention. Figure 4 summarises the individual and mean AKPS scores at each assessment point.

AB1 showed the largest improvement (62–91 points over 6 weeks). EF3 peaked at week 3 then declined. H5 consistently performed best throughout all timepoints. Most groups demonstrated upward trajectories, though the overall group mean plateaued around week 3 with slight decline by week 6.

## Discussion

This study investigated the feasibility and preliminary effects of a hybrid service delivery approach combining face-to-face and TR methods for runners with PFP in South Africa. As the first study examining a TR hybrid approach in this context, it provides valuable insights for healthcare delivery in resource-constrained settings.

The substantially slower recruitment rate compared to international studies, such as De Oliveira Silva et al. (15) in Australia, reveals important contextual differences that extend beyond simple numerical comparisons. While our study achieved its feasibility target, the recruitment challenges likely reflect deeper systemic issues within the South African healthcare landscape, including limited awareness of TR options among both patients and referring practitioners, and potentially different healthcare-seeking behaviours in our population compared to developed countries. Unlike the Australian context where digital health literacy and infrastructure are more established, South African patients may require more extensive education about TR benefits and safety. Future studies should recognize these contextual barriers and invest in comprehensive community engagement strategies, including social media campaigns tailored to local platforms and extensive professional network development, as suggested by Stewart et al. (31).

The perfect adherence rates observed across all participants represent an encouraging initial finding that suggests hybrid TR interventions may be acceptable and manageable once patients engage with the approach. However, given our limited sample size, this finding requires cautious interpretation and validation through larger studies encompassing participants with diverse socioeconomic characteristics, including varying financial resources, educational backgrounds, and income levels, before broader conclusions can be drawn about hybrid TR feasibility in South African healthcare contexts. This preliminary finding is particularly noteworthy given the economic and geographical barriers that typically impede consistent healthcare access in South Africa. While our results align with Lang et al.'s (32) systematic review findings, our context differs substantially—our participants faced infrastructure challenges and potentially lower digital literacy compared to populations in developed countries where most online intervention research has been conducted. The positive compliance metrics, corroborated by Aily et al. (33) and Tore et al. (34), suggest that consistent virtual physiotherapist interaction may help overcome traditional barriers to treatment adherence in resource-constrained settings, though this hypothesis requires testing across more heterogeneous populations to establish generalizability.

The preference for in-person consultations over virtual sessions reveals critical insights into patient expectations and the limitations of current TR delivery in South Africa. While participants demonstrated that virtual consultations were feasible and effective, their comparative preference for face-to-face sessions likely stems from multiple interconnected factors beyond simple technological challenges. The network connectivity issues documented by Nizeyimana et al. (9) and Hasani et al. (35) in similar low-resource settings may create anxiety and frustration that undermines the therapeutic relationship. However, our findings suggest additional considerations: patients may perceive in-person sessions as more “legitimate” or comprehensive, potentially reflecting cultural expectations about healthcare delivery that prioritize physical presence and hands-on assessment. Unlike studies by Fang et al. (36) conducted in technologically advanced settings, our participants’ preferences may be influenced by unfamiliarity with digital healthcare rather than inherent limitations of TR itself.

The substantial clinical improvements observed demonstrate that hybrid TR can achieve meaningful therapeutic outcomes in PFP management, comparable to traditional face-to-face approaches. These results, aligning with Arslan and Gültekin's (13) 6-week study, suggest that the evidence-based exercise protocol established by Leibbrandt & Louw (37) for the South African context translates effectively to hybrid delivery models. This finding is particularly encouraging given concerns that TR might compromise treatment quality in hands-on disciplines like physiotherapy.

The findings of our study collectively suggest that hybrid TR approaches represent a viable strategy for addressing healthcare access inequities in South Africa, though implementation must be carefully calibrated to local contexts. The success of WhatsApp video consultations demonstrates how leveraging familiar technology platforms can reduce barriers to adoption. However, future research should prioritize understanding patient preferences through qualitative exploration, investigating whether preference patterns change with increased TR exposure, and developing implementation strategies that address both infrastructure limitations and patient education needs. The optimal hybrid model for South African healthcare may differ significantly from approaches successful in developed countries, requiring sustained investment in both technological infrastructure and patient digital health literacy, as supported by Seron et al.'s (38) review of TR effectiveness in diverse healthcare.

### Study limitations

Several methodological limitations must be acknowledged. The small sample size (*n* = 5), while appropriate for feasibility assessment, limits generalisability and interpretability of results. This limitation was further compounded by the requirement for participants to attend face-to-face sessions at a single facility, which may have excluded potential participants due to geographical constraints. Future studies should include larger, more geographically diverse samples with control groups to validate these preliminary findings.

The study's generalizability is limited by the absence of socioeconomic data, which prevents an assessment of whether participants represent underserved populations that may respond differently to telerehabilitation interventions. In addition, collecting anthropometric and psychosocial variables (catastrophizing, kinesiophobia) could have provided valuable insights about responders vs. non-responders, generating important hypotheses for future definitive trials.

Recruiting from physiotherapy departments, selected participants who overcame primary access barriers, and limited feasibility assessment in hard-to-reach populations. Future studies should employ community-based recruitment and collect comprehensive baseline data to better represent underserved populations.

The study design presents inherent limitations regarding bias and validity. All outcome measures were self-reported or assessed by an unblinded researcher, raising the possibility of response bias. The absence of randomisation and control groups limits the validity of effectiveness conclusions. Participants might have felt compelled to provide more favourable responses, particularly when self-reporting exercise compliance through home exercise diaries, given that the same researcher conducted all assessments and interactions throughout the study.

Unblinded outcome assessments may have introduced evaluator bias, potentially compromising study objectivity. Future research should employ blinded evaluation or independent assessors to enhance reliability.

The feasibility assessment relied heavily on self-reported measures without objective verification methods. Future studies should incorporate objective adherence monitoring technologies and blinded outcome assessors to minimise bias. Additionally, the study did not assess cost-effectiveness, which is crucial for healthcare policy decisions in resource-constrained settings.

## Conclusion

This feasibility study establishes that hybrid TR represents a viable and clinically effective approach for managing patellofemoral pain in South African runners, demonstrating exceptional adherence rates and meaningful clinical improvements in pain and functional outcomes. The findings reveal critical insights into healthcare delivery adaptations necessary for resource-constrained settings, where patients pragmatically embrace technology-mediated care despite infrastructure limitations and preference for traditional face-to-face consultations.

The successful implementation using widely available WhatsApp technology demonstrates that effective TR depends more on leveraging familiar platforms than introducing sophisticated systems. This approach directly addresses South Africa's healthcare access barriers while respecting patient preferences and working within existing infrastructure constraints. The study's significance extends beyond clinical outcomes, revealing how hybrid models can bridge the gap between healthcare demand and limited specialist availability in developing healthcare systems.

These findings have important implications for healthcare policy and service delivery in similar resource-limited contexts globally. The hybrid model offers a scalable solution that enhances rather than replaces traditional care, potentially transforming rehabilitation accessibility while maintaining quality standards. However, successful implementation requires concurrent infrastructure development and connectivity support to ensure equitable access across all regions. Future research should focus on large-scale randomized controlled trials, cost-effectiveness analyses, and optimal hybrid ratios for diverse populations. This study provides foundational evidence that TR can democratize access to specialized musculoskeletal care in underserved settings, potentially reshaping rehabilitation delivery models in low- and middle-income countries facing similar healthcare challenges.

## Data Availability

The raw data supporting the conclusions of this article will be made available by the authors, without undue reservation.

## References

[1] Xu J Cai Z Chen M Wang X Luo X Wang Y. Global research trends and hotspots in patellofemoral pain syndrome from 2000 to 2023: a bibliometric and visualization study. Front Med. (2024) 11:1370258. 10.3389/fmed.2024.1370258 PMC10985266 38566926

[2] Leibbrandt DC Louw Q. The development of an evidence-based clinical checklist for the diagnosis of anterior knee pain. S Afr J Physiother. (2017) 73(1):1–10. 10.4102/sajp.v73i1.353 PMC6093140 30135903

[3] Fatimah I Waqqar S. Effects of tibiofemoral mobilization in patients of patellofemoral pain syndrome. J Pak Med Assoc. (2021) 71(11):2506. 34783726 10.47391/JPMA.04-585

[4] Biney E Amoateng AY Ewemooje OS. Inequalities in morbidity in South Africa: a family perspective. SSM Popul Health. (2020) 12:100653. 10.1016/j.ssmph.2020.100653 32939393 PMC7476866

[5] Kizony R Weiss PL Harel S Feldman Y Obuhov A Zeilig G Tele-rehabilitation service delivery journey from prototype to robust in-home use. Disabil Rehabil. (2017) 39(15):1532–40. 10.1080/09638288.2016.1250827 28004980

[6] Richmond T Peterson C Cason J Billings M Terrell EA Lee AC American telemedicine association’s principles for delivering telerehabilitation services. Int J Telerehabil. (2017) 9(2):63. 10.5195/ijt.2017.6232 29238450 PMC5716618

[7] Cason J. Telehealth opportunities in occupational therapy through the affordable care act. Am J Occup Ther. (2012) 66(2):131–6. 10.5014/ajot.2012.662001 22394522

[8] Wang Q Lee RL Hunter S Chan SW. The effectiveness of internet-based telerehabilitation among patients after total joint arthroplasty: a systematic review and meta-analysis of randomised controlled trials. J Telemed Telecare. (2023) 29(4):247–60. 10.1177/1357633X20980291 33459120

[9] Nizeyimana E Joseph C Louw QA. Organizational readiness and rehabilitation professionals’ views on integrating telerehabilitation into service delivery and students’ clinical training: a qualitative study. Digit Health. (2023) 9:20552076231212314. 10.1177/20552076231212314 38025095 PMC10631339

[10] Cottrell MA Galea OA O'Leary SP Hill AJ Russell TG. Real-time telerehabilitation for the treatment of musculoskeletal conditions is effective and comparable to standard practice: a systematic review and meta-analysis. Clin Rehabil. (2017) 31(5):625–38. 10.1177/0269215516645148 27141087

[11] Amin J Ahmad B Amin S Siddiqui AA Alam MK. Rehabilitation professional and patient satisfaction with telerehabilitation of musculoskeletal disorders: a systematic review. Biomed Res Int. (2022) 2022:7366063. 10.1155/2022/7366063 35958819 PMC9363217

[12] Lee JH Shin KH Lee GB Son S Jang KM. Comparison of functional outcomes between supervised rehabilitation and telerehabilitation in female patients with patellofemoral pain syndrome during the COVID-19 pandemic. Int J Environ Res Public Health. (2023) 20(3):2233. 10.3390/ijerph20032233 36767600 PMC9915527

[13] Arslan T Gültekin MZ. The effect of a supervised online group exercise program on symptoms associated with patellofemoral pain syndrome in women. Technol Health Care. (2023) 31(2):771–82. 10.3233/THC-220533 36442169

[14] Albornoz-Cabello M Barrios-Quinta CJ Barrios-Quinta AM Escobio-Prieto I Cardero-Durán MD Espejo-Antunez L. Effectiveness of tele-prescription of therapeutic physical exercise in patellofemoral pain syndrome during the COVID-19 pandemic. Int J Environ Res Public Health. (2021) 18(3):1048. 10.3390/ijerph18031048 33504042 PMC7908506

[15] De Oliveira Silva D Pazzinatto MF Crossley KM Azevedo FM Barton CJ. Novel stepped care approach to provide education and exercise therapy for patellofemoral pain: feasibility study. J Med Internet Res. (2020) 22(7):e18584. 10.2196/18584 32706674 PMC7407256

[16] Amirabadi N Hessam M Monjezi S Molhemi F Mehravar M Hosseinpour P. Effectiveness of telerehabilitation intervention to improve pain and physical function in people with patellofemoral pain syndrome: study protocol for a randomized controlled trial. Trials. (2024) 25(1):195. 10.1186/s13063-024-08047-3 38504365 PMC10949657

[17] Costa F Janela D Molinos M Lains J Francisco GE Bento V Telerehabilitation of acute musculoskeletal multi-disorders: prospective, single-arm, interventional study. BMC Musculoskelet Disord. (2022) 23(1):29. 10.1186/s12891-021-04891-5 34983488 PMC8728982

[18] Paskins Z Bullock L Manning F Bishop S Campbell P Cottrell E Acceptability of, and preferences for, remote consulting during COVID-19 among older patients with two common long-term musculoskeletal conditions: findings from three qualitative studies and recommendations for practice. BMC Musculoskelet Disord. (2022) 23(1):312. 10.1186/s12891-022-05273-1 35366845 PMC8976169

[19] Nanda U Luo J Wonders Q Pangarkar S. Telerehabilitation for pain management. Phys Med Rehabil Clin N Am. (2021) 32(2):355. 10.1016/j.pmr.2021.01.002 33814062 PMC9585226

[20] El-Gilany AH. What is case series. Asp Biomed Clin Case Rep. (2018) 1(01):10–5.

[21] Abu-Zidan FM Abbas AK Hefny AI. Clinical “case series”: a concept analysis. Afr Health Sci. (2012) 12(4):557–62. 23515566 PMC3598300

[22] Alghadir AH Anwer S Iqbal A Iqbal ZA. Test–retest reliability, validity, and minimum detectable change of visual analogue, numerical rating, and verbal rating scales for measurement of osteoarthritic knee pain. J Pain Res. (2018) 11:851–6. 10.2147/JPR.S158847 29731662 PMC5927184

[23] Ittenbach RF Huang G Barber Foss KD Hewett TE Myer GD. Reliability and validity of the anterior knee pain scale: applications for use as an epidemiologic screener. PLoS One. (2016) 11(7):e0159204. 10.1371/journal.pone.0159204 27441381 PMC4956048

[24] Watson CJ Propps M Ratner J Zeigler DL Horton P Smith SS. Reliability and responsiveness of the lower extremity functional scale and the anterior knee pain scale in patients with anterior knee pain. J Orthop Sports Phys Ther. (2005) 35(3):136–46. 10.2519/jospt.2005.35.3.136 15839307

[25] Parmanto B Lewis AN Jr Graham KM Bertolet MH. Development of the telehealth usability questionnaire (TUQ). Int J Telerehabil. (2016) 8(1):3–10. 10.5195/ijt.2016.6196 27563386 PMC4985278

[26] Thompson SK. Sampling. New York/Chichester/Brisbane/Toronto/Singapore: John Wiley & Sons (2012).

[27] Jacques RM Ahmed R Harper J Ranjan A Saeed I Simpson RM Recruitment, consent and retention of participants in randomised controlled trials: a review of trials published in the national institute for health research (NIHR) journals library (1997–2020). BMJ Open. (2022) 12(2):e059230. 10.1136/bmjopen-2021-059230 35165116 PMC8845327

[28] Salaffi F Stancati A Silvestri CA Ciapetti A Grassi W. Minimal clinically important changes in chronic musculoskeletal pain intensity measured on a numerical rating scale. Eur J Pain. (2004) 8(4):283–91. 10.1016/j.ejpain.2003.09.004 15207508

[29] Crossley KM Bennell KL Cowan SM Green S. Analysis of outcome measures for persons with patellofemoral pain: which are reliable and valid? Arch Phys Med Rehabil. (2004) 85(5):815–22. 10.1016/S0003-9993(03)00613-0 15129407

[30] Khurram SS Aga IZ Muzzamil M Karim M Hashmi S. Transitioning from crisis to continuity post-COVID-19 pandemic: adoption of telehealth by Sehat Kahani healthcare providers, Karachi, Pakistan. Telehealth Med Today. (2024) 9(6). 10.30953/thmt.v9.541

[31] Stewart AL Nápoles AM Piawah S Santoyo-Olsson J Teresi JA. Guidelines for evaluating the feasibility of recruitment in pilot studies of diverse populations: an overlooked but important component. Ethn Dis. (2020) 30(Suppl 2):745. 10.18865/ed.30.S2.745 33250621 PMC7683033

[32] Lang S McLelland C MacDonald D Hamilton DF. Do digital interventions increase adherence to home exercise rehabilitation? A systematic review of randomised controlled trials. Arch Physiother. (2022) 12(1):24. 10.1186/s40945-022-00148-z 36184611 PMC9527092

[33] Aily JB Barton CJ Mattiello SM Silva DD De Noronha M. Telerehabilitation for knee osteoarthritis in Brazil: a feasibility study. Int J Telerehabil. (2020) 12(2):137. 10.5195/ijt.2020.6323 33520101 PMC7757647

[34] Tore NG Oskay D Haznedaroglu S. The quality of physiotherapy and rehabilitation program and the effect of telerehabilitation on patients with knee osteoarthritis. Clin Rheumatol. (2023) 42(3):903–15. 10.1007/s10067-022-06417-3 36279075 PMC9589787

[35] Hasani F Malliaras P Haines T Munteanu SE White J Ridgway J Telehealth sounds a bit challenging, but it has potential: participant and physiotherapist experiences of gym-based exercise intervention for achilles tendinopathy monitored via telehealth. BMC Musculoskelet Disord. (2021) 22(1):138. 10.1186/s12891-020-03907-w 33541314 PMC7860049

[36] Fang BK Jiang JJ Loh JK Ismail SA. Telerehabilitation acceptance among patients during circuit breaker period: a retrospective study. Dialogues Health. (2022) 1:100049. 10.1016/j.dialog.2022.100049 38515891 PMC10953890

[37] Leibbrandt D Louw Q. The effect of an individualised functional retraining intervention on pain, function and biomechanics in participants with patellofemoral pain: a series of n of 1 trial. J Phys Ther Sci. (2019) 31(1):39–52. 10.1589/jpts.31.39 30774204 PMC6348178

[38] Seron P Oliveros MJ Gutierrez-Arias R Fuentes-Aspe R Torres-Castro RC Merino-Osorio C Effectiveness of telerehabilitation in physical therapy: a rapid overview. Phys Ther. (2021) 101(6):pzab053. 10.1093/ptj/pzab053 33561280 PMC7928601

